# Dynamics of Eye Dominance Behavior in Virtual Reality

**DOI:** 10.16910/jemr.17.3.2

**Published:** 2024-02-28

**Authors:** Franziska Prummer, Ludwig Sidenmark, Hans Gellersen

**Affiliations:** Lancaster University, Lancaster, United Kingdom; University of Toronto, Toronto, Ontario, Canada

**Keywords:** Dominant eye, eye tracking, virtual reality, distance in VR

## Abstract

Prior research has shown that sighting eye dominance is a dynamic behavior and dependent on horizontal viewing angle. Virtual reality (VR) offers high flexibility and control for studying eye movement and human behavior, yet eye dominance has not been given significant attention within this domain. In this work, we replicate Khan and Crawford’s (2001) original study in VR to confirm their findings within this specific context. Additionally, this study extends its scope to study alignment with objects presented at greater depth in the visual field. Our results align with previous results, remaining consistent when targets are presented at greater distances in the virtual scene. Using greater target distances presents opportunities to investigate alignment with objects at varying depths, providing greater flexibility for the design of methods that infer eye dominance from interaction in VR.

## Introduction

Sighting dominance refers to the subconscious preference for one eye over another in tasks that require the alignment of objects at different depths in the visual field (11). Individuals have a preferential tendency for one eye, referred to as eyedness (12). Still, Khan and Crawford (5) have shown that sighting dominance is dynamic and dependent on context, specifically the horizontal gaze angle at which objects are aligned. In this work, we examine sighting dominance in virtual reality (VR) as a technology that affords immersion in 3D simulated environments while relying on binocular fusion of computer-generated images presented separately to each eye. We present a replication of Khan and Crawford’s original study in VR to assess whether their findings hold in VR and extend the experiment to study alignment with objects presented at greater depth in the visual field.

Most tests of sighting eye dominance, such as standard alignment or hole-in-card tests, rely on subjects indicating what they see (11). Subjects are asked to align objects at differing depths, to close one eye alternately, and to report on the perceived alignment. For one of the eyes, the alignment will remain as perceived with both eyes open, and that eye is classified as dominant. Sighting dominance is typically tested at a central viewing angle directly ahead of the participant, eliminating any influence by gaze angle and leading to a popular notion of one eye generally dominating. However, Khan and Crawford (6) demonstrated that crossovers in eye dominance occur when objects are aligned in the contralateral field. Banks et al. (1) suggested this to be explained by the relatively larger image size in the eye that is closer to the object at a given viewing angle

Khan and Crawford (5) adopted a more objective approach to determine the dominant eye. Their method required participants to reach and grasp a ring placed around a target and to move the ring towards their face while continuing to fixate the target through the ring. This method ensured that the ring would be brought up close to the one eye dominating alignment of the target through the ring, enabling the investigator to observe and record eye dominance accordingly. The experiment was conducted in a physical environment with targets located at a distance of 0.53cm to facilitate reach, and placed at different gaze angles, from central viewing at 0° to eccentric viewing at 50° to the left or right, in steps of 10°. For central viewing, eye dominance was influenced by individual differences, but at eccentric angles, it depended consistently on the position in the visual field. Additionally, a hand effect was observed with more left eye dominant cases when the left hand was used to grasp the ring and vice versa, relating to other work on the link between handedness and eyedness (2).

VR has become a valuable tool for studying eye movement and human behavior, as it provides flexibility and control in presentation of stimuli in a 3D virtual environment (7). Variables in VR experiments can be highly controlled, yet the experimental setup may still greatly resemble real-world scenarios that can be replicated with little effort (3). Within a VR scene, the position of objects presented is controlled and available for analyses, while tracking of gaze, head and hand movement affords precise measurements in relation to targets viewed and manipulated. Eye movement is being studied in VR to support interaction (10). Sidenmark and Gellersen (13), for instance, have used VR to study the coordination of eye, head and torso movements in gaze shifts. However, eye dominance has not been given any significant attention. Elbaum et al. (4) considered eye-tracking from the dominant versus the cyclopean eye but assumed a static dominant eye. Meng et al. (8) proposed to optimize foveated rendering by giving priority to the dominant eye, optimizing computing resources without compromising perceived visual quality. This approach assumes consistent eye dominance, but by acknowledging the dynamic nature of eye dominance, there is an opportunity to further refine rendering, ensuring optimal visual quality under varying conditions. Wagner et al. (14) studied gaze-assisted selection in a VR environment by perspective pointing with a finger in the line of sight and found performance to deteriorate when targets were at a greater distance from the finger, indicating the relevance of eye dominance for interactive tasks in VR. Adapting perspective pointing techniques to account for the dynamic changes in individual eye dominance behavior would have the potential to greatly enhance the precision of distance pointing in 3D environments.

In this work, we propose the use of VR for research on eye dominance. We adapt Khan and Crawford’s method for use in a virtual environment and show how this facilitates automated classification of the dominant eye. We replicate the original study on the dependence of eye dominance on gaze angle and hand used to demonstrate that behavior in VR corresponds with behavior in a physical setup. In the original study, only targets that were in reach were used. We take advantage of VR to include targets that are rendered at a greater distance but scaled in size. To the participant, distant targets appear the same size as targets placed in reach, and their alignment action is the same irrespective of target distance (i.e. they do not need to reach any further to find the ring fully surrounding the target). The distance conditions will appear identical in the 2D projection plane but involve increasing disparity in focal depth between ring and target. Our motivation for testing larger distances is to ideally show that the focal disparity does not affect eye dominance, as that would provide greater flexibility for the design of methods that infer eye dominance from interaction in VR.

## Method

We propose using VR to study eye dominance in stereoscopic head-mounted displays (HMD). Modern stereoscopic VR HMDs consist of two displays each providing visual input to only one eye, which creates the experience of a 3D environment. When using VR, users can hold a physical controller that is represented by a visual marker, cursor or object within the VR experience, while the hand itself and any other physical “real-life” surroundings are not. In turn, the HMD prevents surrounding observers from seeing the user’s eyes. If the HMD is equipped with an eye tracker, information about the eyes is available. The eye tracker used in this work (Tobii Pro Research v1.1) provides both monocular information of each eye (position and direction) and cyclopean gaze (origin and direction). For our analysis, we rely on the reported monocular information. The field-of-view (FOV) provided is wider than on conventional displays but narrower than our real vision. The HTC Vive, used in this work, has a 100° horizontal and 110° vertical FOV.

**Figure 1.**
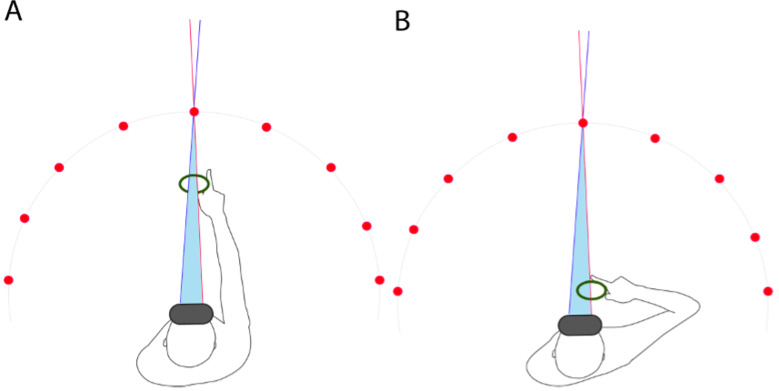
Task Movement. *Note.* (A) The participant focuses on the target and starts by placing the virtual ring around the target. (B) The participant moves the ring closer to their head while continuously fixating on the target through the ring.

In VR, we can determine eye dominance based on tracking of a manually controlled cursor that participants first need to align with a target in the virtual environment, and then move backwards while keeping it aligned with the target, as illustrated in Figure 1. The target locations are fixed to the virtual camera to follow the user’s head movements. This provides control over the gaze angle without need to constrain head movement. VR affords flexibility in the placement of targets. To replicate Khan and Crawford’s work within the limits of the available HMD, we placed targets in range from a -40° to 40°, at 10° increments. In the original study, targets were 3cm in diameter at a 0.53m distance. We implement targets in VR at the respective angular size of 3.2423°. However, we vary the depth at which targets are rendered in the virtual environment to appear at distances of 0.53cm, 1m, and 3m from the viewer. Note, that targets appear at the same perceived distance to the participant, irrespective of their distance.

**Figure 2.**
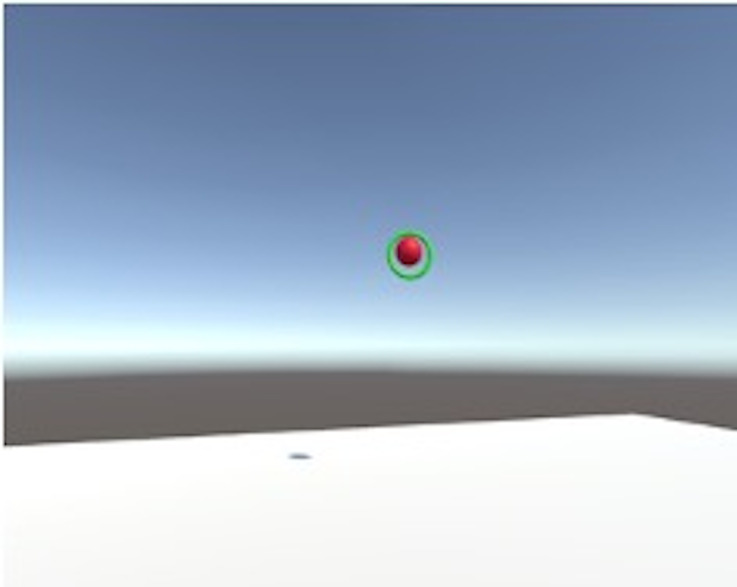
Screenshot of VR task. *Note.* Participant views distant target and aligns the virtual ring around it. The green color confirms the correct placement and signifies the participant to initiate the movement towards the head.

**Figure 3.**
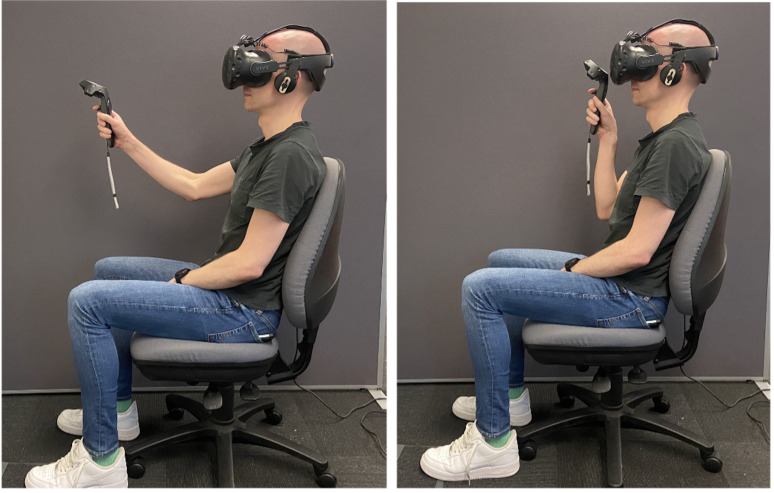
Screenshot of VR task. *Note.* Left: Start of the trial. Right: Completion of the movement.

VR allows flexibility in how cursor and targets are rendered and placed in the environment. However, to replicate Khan and Crawford’s original study, we adopt a ring as the cursor and targets that fit within the ring when they are aligned, as shown in Figure 2. The position of the ring is controlled with a handheld controller, as shown in Figure 3, and continually tracked. An alignment trial starts with a target appearing in the virtual environment. The participant is tasked to align the target by placing the ring around the target. Visual feedback is given by changing the ring color when the participant reaches a preset depth in the virtual environment, set to 53cm to reflect the original setup. The reaching distance is the same in all target conditions, but larger distances induce a focal disparity between the ring and target. To avoid a collision of the controller with the HMD in the backward movement, we also placed a virtual collider just in front of the HMD. Once the virtual ring reaches this collider, a notification sound signals that the trial has been completed.

**Figure 4.**
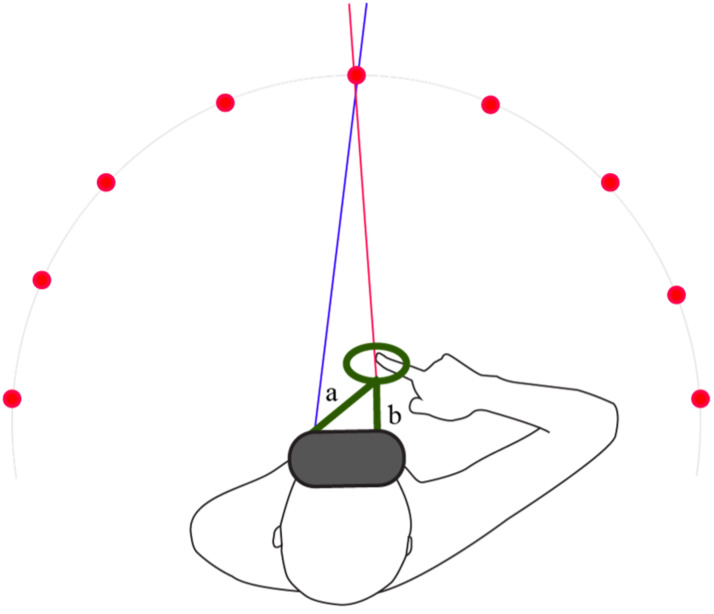
Screenshot of VR task. *Note.* At the end of the movement, the distances of each eye to the ring are compared (a and b). The shorter of the two distances (in this case b) classifies the dominant sighting eye.

In the work by Khan and Crawford (5), the respective eye over which participants placed the ring at the end of the movement was manually labelled as dominant by visual inspection of the video-recorded user. In VR, we can automate the classification. We track the backward movement of the ring and at the end position and measure the distances from either eye (cf. Fig. 4). The eye that is closer to the ring is labelled as the dominant eye. To do this we used eye tracking data provided by the HMD. The eye tracking data was exclusively utilized for trial validation. This approach allowed for an automatic dominant eye classification procedure. Note, since participants are wearing an HMD, they cannot fully reach their eye as the original study did (cf. Fig. 3). The HMD adds 10cm in depth, and in post-hoc analysis, we found that the final distance of the ring from the dominant eye was on average at *M*=12.74cm (*SD*=0.92cm). This close to the face, the difference in distance to either eye is pronounced, providing a robust measure for classification.

## Study

The objective of our study was to replicate Khan and Crawford’s original study on task dependence of eye dominance in VR. In addition, our objective was to test conditions where targets appear at a greater distance from the viewer, to assess whether the focal disparity present at the start of the alignment affects the choice of dominant eye.

### Participants and Apparatus

20 participants were recruited (11 male, 8 female, 1 preferred not to indicate gender, *M* = 31.2 *SD* = 6.68 years) from our local university. Eight participants reported normal vision, eleven corrected to normal vision with glasses, and one corrected to normal vision with lenses. Participants with corrected vision were asked to wear contact lenses instead of glasses for the study. 14 of the participants reported being right- and six left-handed. Six participants were cross-dominant (e.g., left-hand and right-eye dominant, and vice versa). The standard alignment test showed eight participants were dominant in the left eye, while twelve proved dominancy in the right (11).

The study setting and its conditions were created with Unity 2020.3.32f1. An HTC Vive (90Hz display refresh rate) with an integrated Tobii Pro Research v1.1 eye tracker (sample rate 120Hz) was used to record hand controller and eye movements.

### Design and Procedure

The factors studied were viewing angle, target distance and use of left versus right hand:

- Viewing Angle {-40, -30, -20, -10, 0, 10, 20, 30, 40°}
- Target Distance {0.53, 1, 3m}
- Hand used {Left, Right}

As Khan and Crawford reported a hand effect, we also included this as an independent variable. To avoid a bias, the hand used during the trials was counterbalanced with a 3 target distances x 2 hands balanced Latin square. Distinct variable combinations were repeated 5 times, resulting in a total of 270 trials per participant, consisting of 45-trial blocks.

Before participation, subjects gave informed consent. Participants completed a short demographic questionnaire and performed a standard alignment test to determine standard sighting dominance. Subsequently, the task procedure was described, which participants could practice before starting the data collection trials. Before data collection, the participant calibrated the eye tracker with a five-point calibration. Additionally, the inter-pupillary distance was adjusted by rotating the IPD knob on the HMD until the visual indicator in the UI turned green, signifying correct adjustment for optimal depth perception. Participants were instructed to look forward during the study. On average, the study took a total of 45 minutes to complete, with short breaks every 45 trials. The eye tracker was re-calibrated every time participants removed the HMD during breaks. The FST Ethics Committee at Lancaster University ethically approved the study.

## Results

Participants took between 0.85 and 6.34 seconds to complete the movement of each individual trial (*M* = 2.34, *SD* = 0.94 seconds). Some subjects reported experiencing double vision of the ring at farther target distances. This was especially the case in viewing angles located toward the center rather than on the periphery. However, some participants denoted that this did not occur at the outermost target angles (40° and -40°) and saw only a single ring and target. One participant mentioned the inability to see targets located at the outermost viewing angles (-40° and 40°). We discarded the data of this participant from the analysis.

### Data Cleaning

Before analysis, the data was examined for any tracking inaccuracies. Any data frame labelled as “invalid” by the eye tracker was deemed invalid. A single trial was considered valid only if it consisted of at least 90% valid frames. The data of 6 participants was excluded from the analysis, as the collected data of each participant consisted of less than 90% valid trials. Additionally, the remaining 14 participants, a total of 451 individual trials were discarded from the analysis, as these contained less than 90% valid frames. For the remaining trials, we applied backfill linear interpolation. This resulted in a total of 3329 valid trials from 14 participants available for the analyses.

### Binomial Logistic Regression Model

In Khan and Crawford’s (5) study, the final positioning of the ring in front of subjects’ faces was used to indicate the dominant eye. To compare our results to those of Khan and Crawford, we based our analyses on the final data frames of each trial, as these correspond with the final positioning of the ring approximately 10-15cm in front of the subject’s face. This assured the closest imitation of the classification in the original study in VR. The following analyses aim to explore whether our VR findings align with those from the real-life study.

A binomial logistic regression was performed to determine the effects of the angle, distance, and hand used on the probability that participants are right-eye dominant. Table 1 presents the binomial logistic regression model. The model was statistically significant, *𝜒*^2^(3) =3009*.*49, *𝑝*<*.*0005. The model explained 79.20% (9) of the variance in right eye dominance and correctly classified 90.8% of the cases. The sensitivity was 90.40%, the specificity was 91.30%, the positive predictive value was 91.93%, and the negative predictive value was 89.68%. Only two of the three predictor variables were statistically significant: viewing angle and hand used to move the virtual ring (as shown in Table 1). The probability of being right-eye dominant is 5.847 times higher for trials using the right hand than those using the left hand while holding all other variables constant. Right-handed trials were more likely to be right-eye dominant than right-handed individuals. The probability of being right-eye dominant increases by a factor of 1.178 for every 10° increment in angle, while keeping all other variables constant. Therefore, trials with larger angles (>10°) are more likely to be right-eye dominant than those with smaller angles (< -10°). The area under the ROC curve was .963 (95% CI, .957 to .968).

**Table 1.** Binomial Logistic Regression Model.

							95% C.I. for Exp (B)	
	B	S.E.	Wald	df	Sig.	Exp (B)	Lower	Upper
^a^Hand	-1.766	.141	156.110	1	<.001	5.847	4.432	7.713
Angle	.164	.006	748.246	1	<.001	1.178	1.164	1.192
Distance	.067	.060	1.268	1	.260	1.069	.952	1.202
Constant	-.859	.131	43.181	1	<.001	.424		

*Note.* “^a^Hand” is for the right hand compared to the left.

### Target Viewing Angle

**Figure 5.**
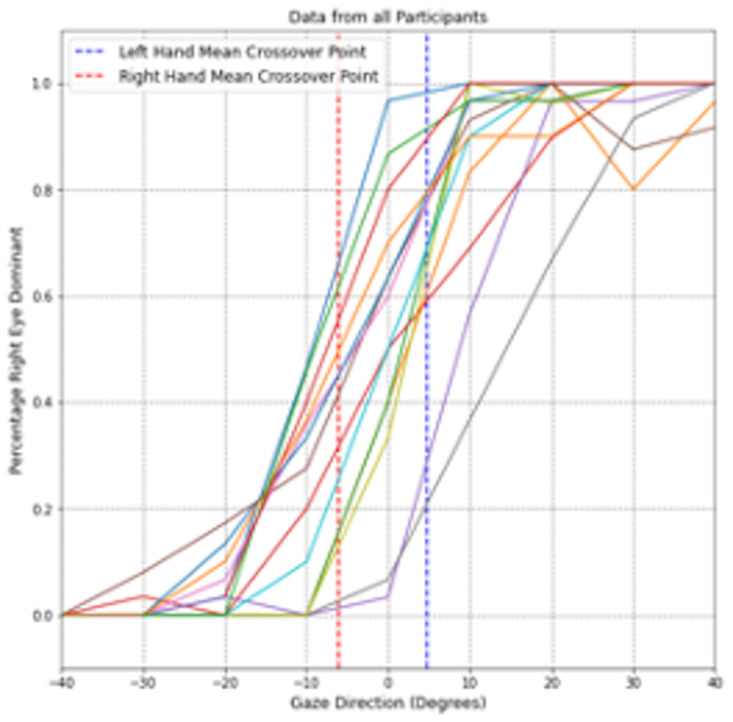
Data of participants’ average percentage of right eye dominant cases of each viewing angle.

Figure 5 visualizes the percentage of a participant being right-eye dominant against each viewing angle for each participant, averaged across all trials and target distances. At the outermost viewing angles, all subjects viewed the targets with the eye corresponding to the respective side. Thus, the targets at -40° were viewed with the left eye, while the subjects used their right eye at 40° viewing angles. On average, when using their right hand, participants switched from their left to their right eye at a gaze angle of around -6.13° (*SD*=9.67), as shown in Figure 5 via the line indicating the mean crossover point. For the left hand, the mean crossover point was located at 4.79° (*SD*=7.80), at which participants switched from their left to right eye.

### Target Distance

**Figure 6.**
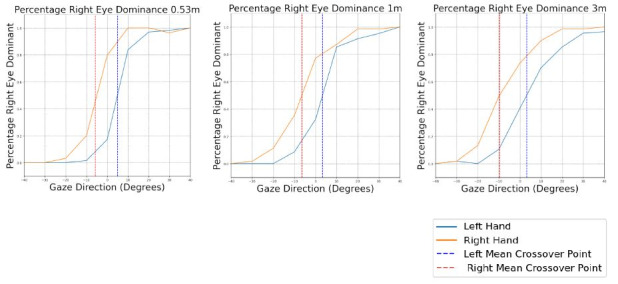
Average percentage of right eye dominant trials against viewing angle for each target distance.

All three target distances display similar trends regarding the percentage of right eye dominant cases against viewing angle (Figure 6). At 0.53m distance, when using their right hand, participants switched from left to right eye at an average gaze angle of –5.74° (*SD*=5.27). At 1m targets, when using the right hand, the left to right eye switch occurs at a mean angle of -6.49° (*SD*=12.44). When viewing targets at 3m and using the right hand, participants switched on average at -9.68° (*SD*=10.60). However, the average viewing angle at which participants switched from left to right eye did not differ significantly with increasing target distance.

### Hand Effect

The influence of the hand's movement on the sighting dominant eye is most pronounced when considering the central viewing angles (-20° to 20°), as depicted in Figure 6. The mean crossover points, the angles at which participants switched from their left to their right eye, are shifted, depending on which hand was used to move the virtual ring. Table 2 presents the individual mean crossover points and respective standard deviations for each target distance and hand used. At targets at 0.53m distance and viewing angle of 0°, 17. 14% of trials are right eye dominant when using the left hand, whereas 79.71% are right eye dominant when using the right hand. At central viewing angles, the right eye is classified more often as dominant, whenever the right hand was used to move the virtual ring. Whenever the left hand moves the ring, the left eye dominates more frequently at the central angles. This shift is independent of target distance.

**Table 2.** Mean crossover points (average angles at which a dominance switch occurs) and standard deviations for “hand” and “distance”.

	0.53m		1m		3m	
	*M*	*SD*	*M*	*SD*	*M*	*SD*
Left	*4.93*	*4.90*	*3.34*	*7.11*	*3.18*	*10.80*
Right	*-5.74*	*5.27*	*-6.49*	*12.44*	*-9.68*	*10.60*

## Discussion

This study showed that the reversal of eye dominance in response to viewing angle applies within a VR setting, remaining consistent when targets are presented at greater depth. Furthermore, we were able to use eye and controller position to determine the dominant eye automatically in the context of a reach and grasp task.

### Eye Dominance Reversal

Our results indicate that the viewing angle and hand used to move a virtual ring to one’s face have a significant effect on sighting eye dominance within a VR context. Target distance does not influence sighting eye dominance significantly. Our results generally agree with those of Khan and Crawford (5), indicating that their main findings apply within a VR setting, even when targets are presented at greater depths. The use of greater target distances presents opportunities to investigate alignment with objects placed at varying depths, providing greater flexibility for the design of methods that infer eye dominance from interaction in VR. However, it is crucial to implement larger target distances carefully, as extending the target distance will result in double vision. Our results indicate a narrower viewing angle range in which an eye dominance switch occurs than Khan and Crawford’s (5). Yet, the large standard deviation of mean crossover points and high variability between participants highlight the individuality in the reversal of sighting eye dominance. When considering the mean crossover points for the left and right hand, the effect of the hand contributing to a switch in eye dominance is highly pronounced. We demonstrated that the close positioning of a virtual ring in front of the face will reliably indicate eye dominance.

### Limitations and Future Work

Several factors limit generalizability of this work. Using the HTC Vive headset limits the horizontal FOV of participants to 100°. With that, targets located at -50° and 50° are not visible, leading to the exclusion of these. Furthermore, this study included the use of static targets that are presented in random order only at a constant horizontal amplitude. It is unclear how target sequencing (e.g., from left to right) or differing target amplitudes would affect eye dominance in VR. This work is also limited by its lack of consideration of head and body rotation. The targets were fixed to the virtual camera, meaning they could rotate their head freely, but the target would still appear at the determined viewing angles. A study taking head and torso position and rotation more closely into account could inspect other factors that cause a shift in eye dominance. Future studies should also investigate the effect of dynamic targets on eye dominance. In addition, further work may develop a “hands-free” approach to classifying the dominant sighting eye. Participants may not be required to use their hands, and a possible influence of hand movement can be discarded. A technique involving two floating targets at different distances may serve as a classification method. In this case, subjects must pivot their head to align both targets. The influence of eye dominance on stereo acuity remains uncertain. However, there is evidence of a bias in the 3D location of objects, with eye dominance being considered a contributing factor (6). Future work should investigate the relationship between eye dominance and stereo acuity, simultaneously examining the participants’ FOV to understand the impact on the virtual experience.

### Conclusion

This work has replicated a real-life study set-up of eye dominance within a VR context. In conclusion, the factors inducing a reversal of sighting eye dominance also apply within VR, remaining consistent when targets are presented at greater distances. Horizontal target viewing angle and the hand used to move a virtual ring towards the face influence a switch in sighting eye dominance. This work is a first step in examining the behavior of eye dominance within VR and using eye and controller position as a means of classification. The results obtained in a VR set-up align with real-world study results.

### Ethics and Conflict of Interest

The authors declare that the contents of the article are in agreement with the ethics described in http://biblio.unibe.ch/portale/elibrary/BOP/jemr/ethics.html and that there is no conflict of interest regarding the publication of this paper.

### Acknowledgements

This work was supported in part by the European Research Council (ERC) under the European Union’s Horizon 2020 research and innovation program (Grant No. 101021229, GEMINI: Gaze and Eye Movement in Interaction).
